# A Double Battle: Fighting Cancer in the Shadows of Conflict in Gaza

**DOI:** 10.7759/cureus.48371

**Published:** 2023-11-06

**Authors:** Abdulqadir J Nashwan

**Affiliations:** 1 Nursing Department, Hamad Medical Corporation, Doha, QAT

**Keywords:** humanitarian crisis, palestine, gaza, conflict, cancer

## Abstract

The ongoing conflict in Gaza has intensified challenges for patients with cancer. Restrictions have limited essential medical supplies, and the recent Israeli airstrike severely damaged Gaza's primary cancer hospital, causing widespread panic. The blockade has caused shortages, and damaged infrastructure has reduced access to care. Many patients seeking treatment outside Gaza face permit delays, and the psychological and economic strains further burden patients and their families. This editorial highlights the broader humanitarian crisis, emphasizing the need for international collaboration to support Gaza's most vulnerable.

## Editorial

The Gaza Strip, a small coastal enclave in the eastern Mediterranean, has been a focal point of geopolitical tensions for decades [1]. The ongoing conflict between Israel and Palestinian factions in Gaza has had profound humanitarian consequences for the region's inhabitants [2]. Even though the recent conflict erupted on the 7th of October, Gaza has been under siege for the past 16 years. Among the most vulnerable are patients with cancer, who face numerous challenges even in peacetime. The current conflict has exacerbated these challenges, making pursuing life-saving treatments even more daunting. As of the 4th of November, the Palestinian Health Ministry reported that the death toll in Gaza has reached 9,488, with 3,900 children and 2,509 women killed, and 24,000 injured [3]. Seventy percent of casualties are vulnerable populations. Additionally, 2,200 are reported missing, and 150 health workers have been killed [3].

The situation for patients with cancer in the Gaza Strip is deeply concerning. According to the Palestinian Ministry of Health, the cancer incidence rate in the region was 91.3 cases per 100,000 people in 2021 [4]. In the same year, there were 1,952 new cancer diagnoses and 610 deaths attributed to the disease [3]. This adds to the cumulative burden of cancer in the area, with a five-year prevalence of cases reaching 10,566 by 2020 [5]. These statistics are set against a backdrop of significant challenges for cancer care in the Gaza Strip, where the ongoing conflict and displacement have led to catastrophic health conditions for patients with cancer. The current conflict has further exacerbated these conditions, placing the lives of approximately 2,000 patients with cancer (on active treatment) at serious risk and contributing to the distress of those living in the Gaza Strip under the duress of Israeli aggression and the resulting displacement [6].

The ongoing conflict has led to severe restrictions on the movement of goods and people into and out of Gaza. This has had a direct impact on the availability of essential medical supplies, including chemotherapy drugs, radiation equipment, and other vital resources for cancer treatment [7]. On the 30th of October, The Turkish-Palestinian Friendship Hospital in Gaza, the largest in Palestine and the only specialized cancer center in Gaza, was severely damaged by Israeli airstrikes [8]. The bombardment has caused panic among patients with cancer and staff. The hospital, funded by Turkey and having 180 beds, now poses increased risks to its patients [8]. On the 3rd of November, four patients with cancer were reported dead after the Turkish-Palestinian Friendship Hospital in Gaza ceased operations due to a fuel crisis [9]. All patients were moved to Dar Al Salam Hospital (10.7 Km on the Southern Gaza Strip and located in Khan Yunis City), as the hospital halted services amidst Israeli attacks and fuel shortages, endangering 70 cancer patients' lives [9]. 

The blockade and intermittent closures of border crossings have resulted in chronic shortages, forcing medical professionals to make difficult decisions about prioritizing patients or even halting treatments altogether. Direct attacks and collateral damage from the conflict have led to the destruction or impairment of medical facilities [10]. Hospitals and clinics, already stretched thin due to the high demand for medical services, face additional strain when parts of their infrastructure are damaged [10]. For patients with cancer, this means reduced access to specialized care units, fewer available beds, and disruptions in ongoing treatments. The destruction of power infrastructure also impacts the preservation of essential medicines and the operation of life-saving equipment.

Due to the limited medical resources in Gaza, many patients with cancer previously sought treatment in neighboring countries or the West Bank [11]. The current conflict has made obtaining permits for medical transfers increasingly difficult. Delays or denials of these permits can result in missed treatment windows, progression of the disease, and, in some tragic cases, preventable deaths.

The psychological impact of living in a conflict zone cannot be overstated [12]. For patients with cancer, the stress of their diagnosis and treatment is compounded by the daily realities of war: the sounds of airstrikes, the fear of sudden displacement, and the grief of losing loved ones. Such chronic stress can have tangible health implications, potentially affecting the efficacy of treatments and the overall prognosis for patients.

The conflict has had a devastating effect on Gaza's economy, leading to high unemployment rates and widespread poverty [13]. For families of patients with cancer, the economic burden of treatment becomes even more pronounced during times of conflict [14]. Many families cannot afford necessary medications, transportation, or supplementary care. Additionally, with many families displaced or fragmented due to the conflict, the support systems that cancer patients rely on can become diminished or non-existent.

The medical community in Gaza, though resilient and dedicated, is not immune to the effects of the conflict [15]. Medical personnel often work in dangerous conditions, facing potential attacks or collateral damage. The constant strain, coupled with the emotional toll of treating an increasing number of trauma patients while also trying to provide care to chronic patients like those with cancer, can lead to burnout, fatigue, and decreased efficacy in patient care [16].

The repercussions of the current conflict on cancer care in Gaza will likely be felt for years to come. The immediate challenges of drugs, damaged infrastructure, and reduced access to specialized treatment have long-term consequences on the overall health outcomes of current patients. Additionally, the disruptions in preventative care, early detection, and routine screenings mean that future cases might be diagnosed at more advanced stages, reducing the chances of successful treatment.

The plight of cancer patients in Gaza is emblematic of the broader humanitarian crisis in the region [17]. While the immediate needs of trauma patients and displaced families are pressing, it's crucial not to overlook the chronic needs of those with severe illnesses like cancer [17]. The international community, humanitarian organizations, and neighboring countries must work collaboratively to ensure that these vulnerable individuals receive the care they need, even in the midst of conflict.

To sum up, the situation in Gaza underscores the tragic reality that in areas of conflict, the most vulnerable often bear the brunt of the suffering. As the world grapples with the broader political and humanitarian implications of the Gaza conflict, it's essential to remember the individual stories of those struggling for survival, like the patients with cancer caught in the crossfire. Their resilience and determination in the face of such adversity serve as a poignant reminder of the human cost of war.
